# A cross-country Exchange Market Pressure (EMP) dataset

**DOI:** 10.1016/j.dib.2017.04.059

**Published:** 2017-05-04

**Authors:** Mohit Desai, Ila Patnaik, Joshua Felman, Ajay Shah

**Affiliations:** National Institute of Public Finance and Policy (NIPFP), New Delhi, India

**Keywords:** Exchange rate regime, Capital flows, Currency wars, Monetary policy, Exchange Market Pressure, Statistical system

## Abstract

The data presented in this article are related to the research article titled - “An exchange market pressure measure for cross country analysis” (Patnaik et al. [1]). In this article, we present the dataset for Exchange Market Pressure values (EMP) for 139 countries along with their conversion factors, ρ (rho). Exchange Market Pressure, expressed in percentage change in exchange rate, measures the change in exchange rate that would have taken place had the central bank not intervened. The conversion factor *ρ* can interpreted as the change in exchange rate associated with $1 billion of intervention. Estimates of conversion factor *ρ* allow us to calculate a monthly time series of EMP for 139 countries. Additionally, the dataset contains the 68% confidence interval (high and low values) for the point estimates of *ρ*’s. Using the standard errors of estimates of *ρ*’s, we obtain one sigma intervals around mean estimates of EMP values. These values are also reported in the dataset.

**Specifications Table**

**Table:** 

Subject Area	Economics, Finance, and Financial economics
More specific subject area	Exchange rates, Exchange market pressure, Currency regime
Type of data	Panel data in long form
How data was acquired	Constructed using methodology outlined in Patnaik et al. [1].
Data format	Analyzed data, empirically derived from other indicators
Experimental factors	–
Experimental features	–
Data source location	http://macrofinance.nipfp.org.in/releases/exchange_market_pressure.html
Data accessibility	Data available with this article and publicly available online on http://macrofinance.nipfp.org.in/releases/exchange_market_pressure.html

**Value of the data**

• The dataset provides consistent estimates of Exchange Market Pressure which can be used to do comparisons across time, as well as across countries.
• Accurate estimates of EMP that can be compared across countries allow researchers to empirically assess the impact of events which affect a set of countries. This could benefit researchers in empirically assessing and comparing the impact of policies followed by different countries.
• EMP provides a sophisticated empirical tool to assess the impact of global or regional events on a set of countries.
• The conversion factor rho enables a clearer understanding of the impact of central bank interventions.

## 1 Data

The dataset described in this article is a long form panel dataset for monthly EMP and *ρ* (conversion factor) values for 139 countries, along with their associated 68% confidence interval values. EMP values are expressed in terms of percentage change in exchange rate while rho values can be interpreted as change in exchange rate associated with $1 billion of intervention by the central bank.

Table 1 provides a glimpse of the EMP dataset. The data shown in Table 1 pertains to United Arab Emirates (UAE) - identified by its two letter code (AE). The two letter code can be mapped to the country name using the file “country_code_map.csv” attached with this article. The column “curr.emp” lists the monthly EMP values and column “rho” provides the value for *ρ* (conversion factor) for the country. For example, the value of 0.11 for January 2001 means that the UAE Dirham was under pressure to appreciate by 0.11%. The negative sign here indicates pressure to appreciate. The value of *ρ* of 4.82 for UAE for 2001 means a central bank purchase of a billion dollars would prevent a 4.82% appreciation in the exchange rate of UAE. The dataset also contains one standard deviation confidence intervals for the mean estimates of EMP and the conversion factor *ρ*.

**Table 1:** EMP dataset.

Date	Country	Curr.emp	emp lo	emp hi	rho	rho lo	rho hi
2001-01-01	ae.curr	−0.11	−0.14	−0.09	4.82	2.20	10.56
2001-02-01	ae.curr	−0.72	−0.87	−0.56	4.82	2.20	10.56
2001-03-01	ae.curr	−0.97	−1.18	−0.77	4.82	2.20	10.56
2001-04-01	ae.curr	−0.32	−0.39	−0.25	4.82	2.20	10.56
2001-05-01	ae.curr	−0.39	−0.47	−0.31	4.82	2.20	10.56
2001-06-01	ae.curr	−0.11	−0.14	−0.09	4.82	2.20	10.56
2001-07-01	ae.curr	0.64	0.50	0.77	4.82	2.20	10.56
2001-08-01	ae.curr	−0.98	−1.19	−0.77	4.82	2.20	10.56
2001-09-01	ae.curr	−1.62	−1.97	−1.28	4.82	2.20	10.56
2001-10-01	ae.curr	−1.88	−2.28	−1.48	4.82	2.20	10.56

This table shows the first ten values of the EMP dataset which is a long form panel dataset for monthly EMP and *ρ* values for 139 countries along with their 68% confidence intervals.

The data attached with this article contains two files – “EMP_all countries.csv” contains the time series of EMP and ρ values and their associated 68% confidence intervals, and “country_code_map.csv” maps the two letter country code with their country names. The dataset has been constructed by using the methodology described in Patnaik et al. [1].

## 2 Experimental design, materials and methods

**Fig. 1:**
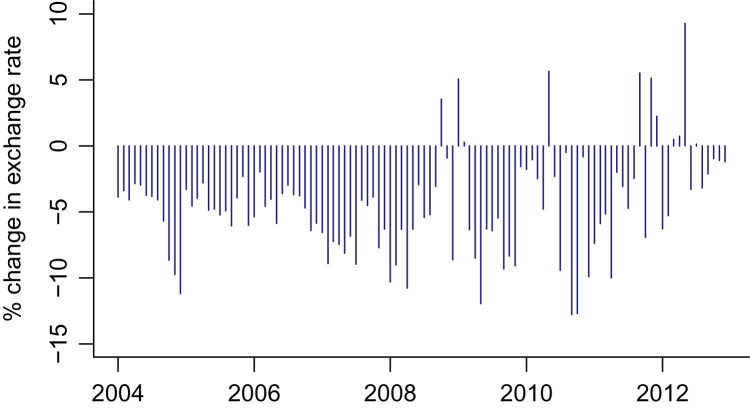
EMP measure for China.

**Fig. 2:**
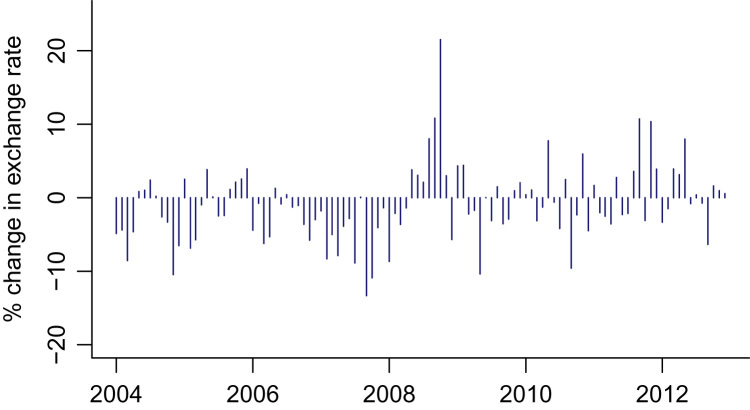
EMP measure for India.

To visualize the dataset, we plot the time-series of EMP values for China and India. Fig. 1, Fig. 2 plot the monthly time-series of EMP values from 2004 to 2013 for China and India, respectively.

**Fig. 3:**
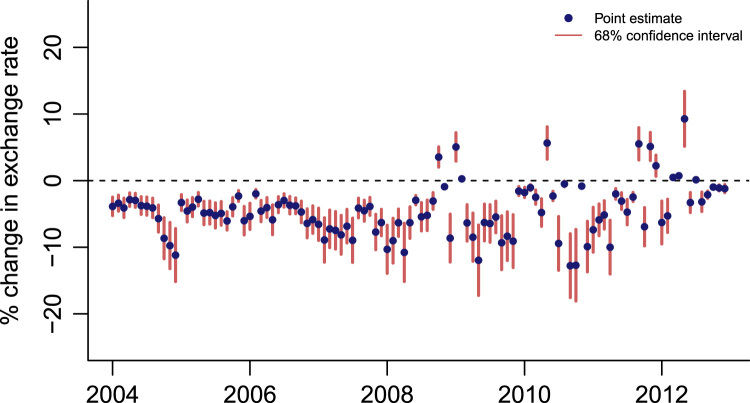
EMP measure for China with confidence intervals.

**Fig. 4:**
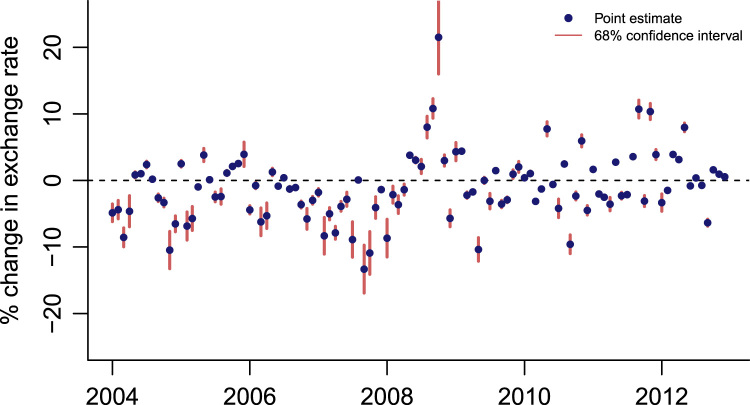
EMP measure for India with confidence intervals.

The 68% confidence intervals for EMP values in our dataset provide a sense of accuracy of our point estimates of EMP. The confidence intervals have been estimated by using the standard errors of ρ values to simulate values of EMP. In Fig. 3, Fig. 4 we plot the point estimates of EMP values along with their 68% confidence intervals for China and India, respectively.

The construction of the EMP dataset (refer Patnaik et al. [1] for the methodology) employs data on exchange rates, GDP, trade, and foreign exchange reserves. These data have been sourced directly from Datastream. The EMP dataset has been coded in the open source language R, and parts of the code – along with the full dataset – can be accessed at:

http://macrofinance.nipfp.org.in/releases/exchange_market_pressure.html

## References

[1] Patnaik I., Felman J., Shah A. (2017). An exchange market pressure measure for cross country analysis. J. Int. Money Financ..

